# Safe deep reinforcement learning in diesel engine emission control

**DOI:** 10.1177/09596518231153445

**Published:** 2023-02-17

**Authors:** Armin Norouzi, Saeid Shahpouri, David Gordon, Mahdi Shahbakhti, Charles Robert Koch

**Affiliations:** Department of Mechanical Engineering, University of Alberta, Edmonton, AB, Canada

**Keywords:** Machine learning, deep learning, reinforcement learning, safe learning, iterative learning control, diesel engine, emission control

## Abstract

A deep reinforcement learning application is investigated to control the emissions of a compression ignition diesel engine. The main purpose of this study is to reduce the engine-out nitrogen oxide $\left(NO_{x}\right)$ emissions and to minimize fuel consumption while tracking a reference engine load. First, a physics-based engine simulation model is developed in GT-Power and calibrated using experimental data. Using this model and a GT-Power/Simulink co-simulation, a deep deterministic policy gradient is developed. To reduce the risk of an unwanted output, a safety filter is added to the deep reinforcement learning. Based on the simulation results, this filter has no effect on the final trained deep reinforcement learning; however, during the training process, it is crucial to enforce constraints on the controller output. The developed safe reinforcement learning is then compared with an iterative learning controller and a deep neural network–based nonlinear model predictive controller. This comparison shows that the safe reinforcement learning is capable of accurately tracking an arbitrary reference input while the iterative learning controller is limited to a repetitive reference. The comparison between the nonlinear model predictive control and reinforcement learning indicates that for this case reinforcement learning is able to learn the optimal control output directly from the experiment without the need for a model. However, to enforce output constraint for safe learning reinforcement learning, a simple model of system is required. In this work, reinforcement learning was able to reduce $NO_{x}$ emissions more than the nonlinear model predictive control; however, it suffered from slightly higher error in load tracking and a higher fuel consumption.

## Introduction

Heavy-duty and medium-duty diesel engines are commonly used for public transportation and delivering goods. The high combustion efficiency and fuel conversion efficiency advantages (especially at full-load operation) along with the long lifetime and durability of diesel engines have made their usage widespread in a wide range of transportation applications.^1,2^ Despite all the advantages, diesel engines contribute significantly to air pollution worldwide. Although hybridization and electrification are getting increasing market share for passenger vehicles, it is expected that this will occur more slowly for heavy-duty applications due to the limited battery range, high battery costs, and increased total cost of ownership.^3^ Therefore, strategies to minimize the effect of diesel engine emissions on the environment are still needed.

Traditionally, engine control units (ECUs) use a feedforward controller that is based on two-dimensional look-up tables, also known as calibration maps, that are generated on a test bench to ensure the engine performs optimally while meeting power demand and enhancing fuel economy, and durability. Additionally, engine emissions are also evaluated during the creation of the these calibration maps making the calibration complicated and time-consuming. These tables must then be further tested in real driving conditions to meet new real driving emission legislation.^1^ The use of a feedback controller, especially a model-based optimal controller, is a promising method to help solve the ever-increasing calibration efforts. Model-based methods such as linear quadratic regulator (LQR),^4^ sliding mode controller (SMC),^5,6^ adaptive,^7,8^ and model predictive control (MPC)^9–11^ have been previously investigated for engine applications. The two main drawbacks of these model-based controllers are their sensitivity to model accuracy and the required runtime especially for online optimization. There is often a trade-off between these two as improving model accuracy requires increased model complexity and these complex models usually exhibit nonlinear behavior requiring a more complicated control law such as nonlinear model predictive controller (NMPC).^12^ Instead of using a model-based controller, the alternative option is using a model-free controller. Machine reinforcement learning (RL)^13^ is one of the powerful methods in generating optimal options without the requirement of the model. Another well-known model-free controller in control theory is iterative learning controller (ILC).^14^ As ILC is also a model-free learning-based control strategy, it will be compared with both RL and a model-based state-of-the-art deep learning–based MPC.

Machine learning (ML) is a powerful tool that has been used to address various engineering problems and has been shown to be particularly useful in control engineering, especially when deriving an exact system model is difficult.^15^ In general, supervised learning, unsupervised learning, and RL are the main categories of ML. Unlike supervised and unsupervised learning that operate using a static data set, RL works using dynamic data.^16^ The main goal of RL is generating the optimal outcome by finding the best sequence of actions. Unlike classical ML, RL uses an agent to explore, interact with, and learn from the defined system environment. The RL agent learns by receiving the environment observation and reward and generating a sequence of actions to reach a specific goal. RL has a similar structure to control theory. The goal is to determine the correct inputs into a system that would generate the desired system’s behavior. The controller is called the policy, the actuator command provides the actions, and the plant is the environment in RL. As we tune the controller using a tuning algorithm or adaptation law, the RL policy updates are based on the RL algorithm.^17^ The RL algorithm can be either model-free or model-based, and due to the model requirement, the model-free algorithm has been the main focus in engineering applications.^16,17^ One common algorithm used for model-free RL is Q-learning. In Q-learning, the value of an action for a particular state is learned and the optimal policy is found by maximizing the expected value (Q-value) of the total reward.^13^

When an agent performs an action which has the highest reward without further exploring the environmental space, it is considered a greedy policy. In continuous spaces, obtaining a greedy policy to optimize the action at each time interval is extremely slow. Therefore, sometimes, it is not possible to apply Q-learning easily to continuous action systems. However, an actor-critic method based on the deterministic policy gradient (DPG) algorithm is a suitable choice for a system with a continuous space.^18^ The DPG learning procedure is robust and stable because of the off-policy network training; it takes samples from the replay buffer (which is a finite size cache used to store previous samples from the environment). This allows for the reduction of the correlation between samples.^19^ Off-policy learning is independent of the agent’s actions and it determines the optimal policy regardless of the agent’s motivation. It means that in contrast with on-policy learning, where the agent learns about the policy to generate the data, the off-policy estimates the reward for future actions and adds value to the new state without following any greedy policy.^13^ The deep deterministic policy gradient (DDPG) agent is a model-free and off-policy RL algorithm where an actor-critic RL agent calculates an optimal policy by maximizing the long-term reward. One of the differences between DDPG and DPG is that DDPG uses a deep neural network (DNN) as an approximator in DDPG to learn for large state and action pairs.^19^

Using DNN in RL is referred to as deep reinforcement learning (deep RL) and has allowed for a wide variety of complicated decision-making tasks that were previously unfeasible to be solved.^13^ For example, deep RL is of interest in applications such as robotics and autonomous driving.^20^ Earlier versions of RL algorithms had challenges in the design of the feature selection. In contrast, deep RL has been able to successfully overcome complicated tasks even when a limited amount of previous information is available. This is possible because of the deep RL capability to learn various levels of abstractions from data.^20–23^ Deep RL has also been used in computer science for many applications.^13^ Utilizing deep RL in real-world applications, especially in engineering applications, has started to increase in recent years. Deep RL has been successfully used for control of an unmanned aerial vehicle,^24^ quadrotor system,^25^ autonomous vehicles,^26,27^ wind farm control,^28^ torque distribution of electric vehicles,^29^ and robotic applications.^30,31^

RL has been used for automotive powertrain control systems especially in energy management of hybrid electric vehicles^32–34^ and for internal combustion engines.^35–40^ Q-learning RL is used as idle speed control of a spark-ignition (SI) engine by controlling the spark timing and intake throttle valve position.^41^ Similar studies have been carried out for diesel engine idle speed control by controlling the fuel injection timing.^36^ RL has also been used for emission control of SI engines.^37,38^ A very limited number of studies have been carried out utilizing RL for internal combustion control, and most of the existing work has focused on SI engines. To the authors’ knowledge, deep RL algorithms have not been previously implemented for diesel engine performance and emission control. Safety concerns and constraint violations of pure learning controllers in highly complex systems such as internal combustion engines have hindered the development of these learning controllers. Fortunately, recent studies have addressed output constraint enforcement in the learning-based controller using a safe learning filter. This method enforces the output constraints and provides a method to implement safe learning RL.^42–45^ To implement the safety filter, a simplified version of a second optimization-based method to enforce output constraints is used. Instead of an MPC-based filter, an online optimization with a single-step optimization is used where the safe control action minimizes the deviation from the RL-generated control action subject to constraints determined using a quadratic programming (QP) solver during the training of the RL agent. Then, the RL agent must learn the constraints using the RL algorithm and prior knowledge of system constraints.

Although RL is now receiving attention from the control system community, a learning controller is not a new concept.^14,46^ One of the well-known learning-based controllers is ILC which is used to improve the tracking performance of a system in the presence of repetitive input or disturbances.^47–49^ ILC was first introduced in 1984 by Arimoto et al.^14^ and since then has been used for various control problems. ILC has a simple structure and is computationally efficient for real-time applications and can have stability guarantees. Different types of ILC have been implemented for internal combustion engine control. ILC has been used in SI engine load control,^50,51^ a dual-fuel control of homogeneous charge compression ignition (HCCI) engine,^52^ SI engine speed and air-to-fuel ratio,^53^ parameter optimization in a turbocharged SI engine,^54^ variable injection rate control for compression ignition (CI) engines,^55^ diesel $NO_{x}$ control,^56,57^ and exhaust gas recalculation (EGR) control in a CI engine.^58^ Although ILC has been used in literature as a model-free learning-based controller, it requires a repetitive environment to learn from the repetition. This is not feasible in an on-road vehicle applications as the engine usually operates under changing conditions. Diesel engines are often used in stationary applications where repetitive operation can occur, for example, used to power a pump or generate power. In those applications, using ILC seems promising.^56,57^ Here, due to the similarity of the ILC concept to RL, and as it has been used in literature for emission control, it has been implemented in simulation and compared with the designed safe deep RL.

Safe learning in the content of deep RL used to control diesel engine emissions is not available in the literature. Therefore, a deep RL with and without safety filters is designed and compared to address this gap. Then to compare RL to ILC, ILC and safe ILC are also designed. Additionally, RL is compared with a deep recurrent neural network–based nonlinear model predictive controller that has been developed in our previous study.^59^ The main contributions of this article are as follows:

- Design of a deep RL controller for diesel engine $NO_{x}$ control by minimizing $NO_{x}$ and fuel consumption while maintaining the same output torque;
- Design of a safe filter that provides safe RL for diesel engine emission control;
- Comparison with a classical learning-based control, ILC, and a long-short-term memory-based nonlinear model predictive controller (LSTM-NMPC^59^).

This article is organized into five sections. The first section provides an introduction, literature review, and main contributions of this article. In the “Engine simulation model” section, the experimental setup and detailed physics-based modeling are explained. The main methodology of the safe deep RL are discussed in section “Deep RL.” Details regarding the development of the ILC are explained in section “ILC.” The “Results and discussions” section illustrates the performance of designed controllers and provides a comparison of the controllers. Finally, the main conclusions of this article are summarized in section “Summary and conclusion.”

## Engine simulation model

This study uses a 4.5-L diesel engine manufactured by Cummins and is located in the advanced internal combustion engine lab at the University of Alberta, Canada. The main specifications of this engine are presented in Table 1.

**Table 1.** Engine specifications.

Parameter	Value
Engine type	In-line, four-cylinder
Displacement	4.5 L
Bore × Stroke	102 mm × 120 mm
Peak torque	624 N m @ 1500 r/min
Peak power	123 kW @ 2000 r/min
Aspiration	Turbocharged
Certification level	Tier 3/Stage IIIA

To train the deep RL used in this study and to compare with NMPC and ILC, a detailed physical model (DPM) was developed in GT-Power software and validated using experimental data in our previous studies.^60,61^ This DPM is implemented in GT-Power and the model includes several physical and chemical sub-models for simulating the combustion phenomenond gas exchange process of diesel combustion. The DPM was calibrated using experimental in-cylinder pressure, injection timing, and intake air mass flow and temperature over the various engine operating range. Optimal parameter values are determined by means of the genetic algorithm (GA). Additional details of the DPM development and structure are presented in the authors’ previous works.^60,61^

**Figure 1.**
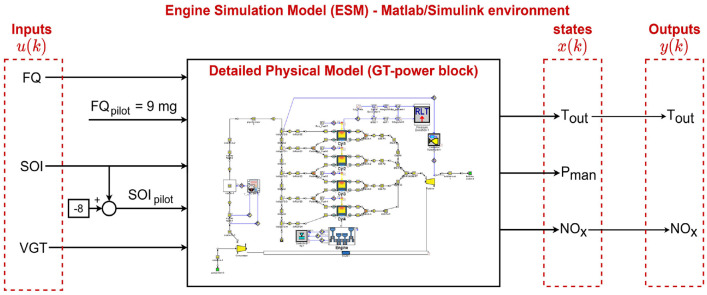
Input, output, and states of engine simulation model (ESM) to simulate engine torque $\left(T_{\mathrm{out}}\right)$ , intake manifold pressure $\left(P_{\mathrm{man}}\right)$ , $NO_{x}$ emissions.

The developed DPM predicts the experimental in-cylinder pressure over the entire engine cycle (see Figures 5 and 6 in the work by Shahpouri et al.^60^) with the maximum in-cylinder pressure and intake manifold pressure error of ±5.8% and ±4.6% . To model $NO_{x}$ emissions, a physical-based model was developed and added to the DPM with accuracy of ±18.1% . This model is parameterized by minimizing the error between the experimental $NO_{x}$ and model prediction of $NO_{x}$ from the model. In this work, the DPM is modeled using GT-Power, and using Matlab/Simulink and GT-Power co-simulation, controller is implemented. This GT-Power/Matlab/Simulink co-simulation called engine simulation model (ESM) and it will be used to test the developed concepts in simulation and the most promising methods will be then reported for real-time implementation in future work. The main inputs and outputs of ESM are schematically shown in Figure 1. The outputs of this model are $NO_{x}$ emissions, output torque $\left(T_{\mathrm{out}}\right)$ , intake manifold pressure $P_{\mathrm{man}}$ , and the inputs of this model are start of injection (SOI) for main diesel fuel injection, fuel quantity (FQ), and variable geometric turbine (VGT) rate. For the sake of simplicity, the start of pilot injection is kept 8°CA before the main injection with a constant FQ of 9 mg per cycle.

## Deep RL

### DDPG agents algorithm

A DDPG agents algorithm is used to minimize the engine-out emissions and fuel consumption while maintaining the same load. DDPG is a model-free and off-policy RL algorithm where an actor-critic RL agent calculates an optimal policy by maximizing the long-term reward. When a DNN is used, the DDPG algorithms are referred to deep DPG. The psudo code of DDPG is shown in Algorithm 1.^19^ During training, the actor and critic are updated by the DDPG algorithm at each sample time, and the agent stores past experiences using an experience buffer. The actor and critic are then updated using a mini-batch of those experiences randomly sampled from the buffer. Also, the policy’s selected action is perturbed using a stochastic noise model at each training step.^17^

**Table:** 

**Algorithm 1:** Deep deterministic policy gradient agents (DDPG) algorithm^19^
Initialize critic network randomly $Q(x,u|\theta^{Q})$ with weights $\theta^{Q}$ Initialize actor network randomly $\mu (x,u|\theta^{\mu })$ with weights $\theta^{\mu }$ Initialize target network $Q^{\prime }$ and $\mu^{\prime }$ with weights $\theta^{Q^{\prime }}\leftarrow \theta^{Q}$ and $\theta^{\mu^{\prime }}\leftarrow \theta^{\mu }$ Initialize replay buffer R **for***episode* =1 , $E_{f}$ **do** Initialize a random noise process N to add action exploration Receive initial observation state x(1) **for** k=1 , $k_{f}$ **do** Select action $a_{t}=\mu (x(k)|\theta^{\mu })+N(k)$ Execute action u(k) and observe reward r(k) and observe new state x(k+1) Store (x(k),u(k),r(k),x(k+1)) in R Sample a random mini-batch of N transition (x(k),u(k),r(k),x(k+1)) from R Set $\hat{r}(k)=r(k)+\gamma Q^{\prime }(x(k+1),\mu^{\prime }(x(k+1)|\theta^{\mu^{\prime }})|\theta^{Q^{\prime }})$ Update critic by minimizing the loss: $L=\frac{1}{N}\Sigma_{i}(\hat{r}(k)-Q(x(k),u(k)|\theta^{\theta^{Q}}){)}^{2}$ Update actor based on the sampled policy gradient: $\begin{array}{c} \nabla_{\theta \mu }J\approx \frac{1}{N}\Sigma \nabla_{a}Q(x,u|\theta^{Q}){|}_{x(k),\mu (x(k))}\nabla_{\theta \mu }\mu (s|\theta^{\mu }){|}_{x(k)} \end{array}$ Update the target network: $\theta^{Q^{\prime }}\leftarrow \tau \theta^{Q}+(1-\tau )\theta^{Q^{\prime }}$ $\theta^{\mu^{\prime }}\leftarrow \tau \theta^{\mu }+(1-\tau )\theta^{\mu^{\prime }}$ **end** **end**

In the DDPG algorithm (Algorithm 1), first, a copy of the actor $Q^{\prime }(x,u|\theta^{Q^{\prime }})$ and the critic network $\mu^{\prime }(x,u|\theta^{\mu^{\prime }})$ has been created. Then, these target network weights are updated “gently” to follow the learned networks: $\theta^{\prime }\leftarrow \tau \theta +(1-\tau )\theta^{\prime }$ with τ<<1 . The target value is constrained to change at a slow rate to improve the stability of learning. Exploration is a significant challenge of learning when the action spaces are continuous. As exploration is an off-policy algorithm, such as DDPG, independent of the learning algorithm, exploration policy $\mu^{\prime }$ can be formed by combining a noise process N with the actor policy. In the DDPG algorithm, the Ornstein–Uhlenbeck process noise model is used to create a noise process for agent exploration.^17,62,63^

### Safe DDPG

Despite all the advantages of deep RL, it relies on the experience and interaction with the environment (here ESM). To enforce output constraints, the following optimization-based filter is added to DDPG algorithm

(1) $$\begin{array}{c} \underset{x}{\mathrm{Minimize}:}\;||u(k)-u_{\mathrm{RL}}(k)|{|}_{2}^{2}\\ \mathrm{subject}\;\mathrm{to}:\;y(k)<y_{\max}\\ \;\;\;\;\;\;\;\;\;\;\;\;\;\;\;u_{\min}<u(k)<u_{\max} \end{array}$$

where u(k) is a safe action and $u_{\mathrm{RL}}(k)$ is the DDPG-generated action. The goal of this optimization is to enforce that the output does not exceed the defined output maximum value $y_{\max}$ given lower $\left(u_{\min}\right)$ and upper bound $\left(u_{\max}\right)$ of actions while minimizing the difference between the DDPG-generated action and the safe action. The optimization of equation (1) uses QP to find the control action u(k) that minimizes the function $||u(k)-u_{\mathrm{RL}}(k)|{|}_{2}^{2}$ . The QP solver applied the following constraints to the optimization

(2) $$\begin{array}{c} f(x(k))+g(x(k))u(k)<y_{\max}\\ u_{\min}<u(k)<u_{\max} \end{array}$$

where f(x(k)) and g(x(k)) are coefficients of the constraint function which depend on the modeled plant states x(k) . Linear plant dynamics developed in our previous study are used in the optimization^11^

(3) $$\begin{array}{c} x(k+1)=\mathrm{Ax}(k)+\mathrm{Bu}(k)\\ y(k)=\mathrm{Cx}(k) \end{array}$$

where A and B are state-space matrices developed using a autoregressive with extra input (ARX) model^11^ as follows

(4) $$\begin{array}{c} A=\left[\begin{array}{ccc} 0.7286 & 7.1252 & -0.0019\\ 0.0002 & 0.9859 & 8.9878\times 10^{-6}\\ -0.6105 & 33.94287 & 0.9076 \end{array}\right]\\ B=\left[\begin{array}{ccc} 1.2639 & -1.0899 & 1.0084\times 10^{-5}\\ -0.0007 & 0.0014 & -1.01397\times 10^{-5}\\ 2.9360 & -8.2453 & -0.0106 \end{array}\right]\\ C=\left[\begin{array}{ccc} 1 & 0 & 0\\ 0 & 0 & 1 \end{array}\right] \end{array}$$

where the constrained output y(k) , states x(k) , and control actions u(k) are defined as follows

(5) $$\begin{array}{c} y(k)=[NO_{x}(k)P_{\mathrm{man}}(k)T_{\mathrm{out}}(k{)]}^{T}\\ y(k)=[NO_{x}(k)T_{\mathrm{out}}(k{)]}^{T}\\ u(k)=[\mathrm{FQ}(k)\mathrm{SOI}(k)\mathrm{VGT}(k{)]}^{T} \end{array}$$

where FQ(k) , SOI(k) , and VGT(k) are injected FQ, start of main injection, and variable-geometry turbocharger (VGT) valve rate (percentage opening), respectively. The states are defined as engine-out $NO_{x}(k)$ emission, intake manifold pressure $P_{\mathrm{man}}(k)$ , and output torque $T_{\mathrm{out}}(k)$ . By substituting equation (3) in equation (2), f(x(k)) and g(x(k)) can be found as follows

(6) $$\begin{array}{c} f(x(k))=\mathrm{CAx}(k)\\ g(x(k))=\mathrm{CB} \end{array}$$

Substituting system matrices (equation (4)) in equation (6) results in the following

(7) $$\begin{array}{c} f(x(k))=\left[\begin{array}{ccc} 0.7286 & 7.1252 & -0.0019\\ -0.6105 & 33.94287 & 0.9076 \end{array}\right]x(k)\\ g(x(k))=\left[\begin{array}{ccc} 1.2639 & -1.0899 & 1.0084\times 10^{-5}\\ 2.9360 & -8.2453 & -0.0106 \end{array}\right] \end{array}$$

To simplify the control problem, the pre-injection is kept constant at 9 mg that is injected 8°CA before the main injection.

The upper bound of $NO_{x}$ is used to regulate peak $NO_{x}$ engine-out emission levels. This value depends on government legislation limits. Here, the experimental maximum $NO_{x}$ level of 500 ppm is observed for the production Tier 3 engine during standard operation load range and this value is used as the upper bound of engine-out $NO_{x}$ . To avoid high loads beyond the defined operating range, a 500 N m torque is used as the upper bound for load. To regulate the amount of injected fuel and avoid large fuel injections, a constraint is imposed for injected fuel amount of 10 to 90 mg/cycle. To avoid late injections that cause combustion inefficiency and high exhaust gas temperatures, a lower limit of SOI is also imposed. Due to the physical limitations, the VGT is limited between 70% and 100%. To avoid increased combustion noise and causing low combustion efficiency, SOI is also limited using an upper bound. Therefore, the constraints can be summarized as follows

(8) $$\begin{array}{cc} & y_{\min}=\;{\left[\begin{array}{cc} NO_{x,\min}(k) & T_{\mathrm{out},\min}(k) \end{array}\right]}^{T}\;=\;{\left[\begin{array}{cc} 0 & 0 \end{array}\right]}^{T}\\ & y_{\max}=\;{\left[\begin{array}{cc} NO_{x,\max}(k) & T_{\mathrm{out},\max}(k) \end{array}\right]}^{T}\;=\;{\left[\begin{array}{cc} 500 & 500 \end{array}\right]}^{T}\\ & u_{\min}=\;{\left[\begin{array}{ccc} FQ_{\min}(k) & \mathrm{SO}I_{\min}(k) & \mathrm{VG}T_{\min}(k) \end{array}\right]}^{T}\;=\;{\left[\begin{array}{ccc} 10 & -2 & 70 \end{array}\right]}^{T}\\ & u_{\max}=\;{\left[\begin{array}{ccc} FQ_{\max}(k) & \mathrm{SO}I_{\max}(k) & \mathrm{VG}T_{\max}(k) \end{array}\right]}^{T}\;=\;{\left[\begin{array}{ccc} 90 & 11 & 100 \end{array}\right]}^{T} \end{array}$$

**Figure 2.**
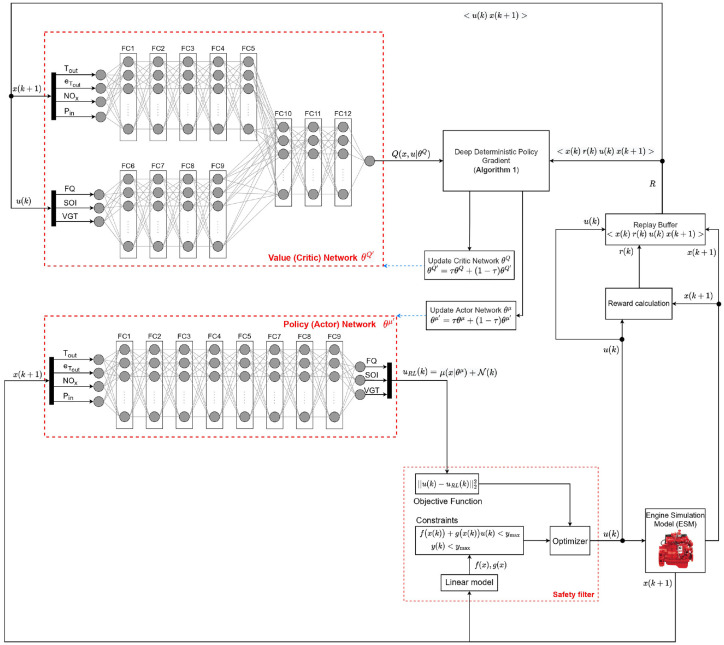
Safe deep deterministic policy gradient schematic to minimize diesel engine fuel consumption and reducing $NO_{x}$ while maintaining the required output torque. FC: fully connected layer.

A schematic of safe DDPG for minimizing diesel engine emissions and fuel consumption while maintaining load is shown in Figure 2. The states of the system for the DDPG algorithm are defined as follows

(9) $$\begin{array}{c} x(k)=[NO_{x}(k)e_{T_{\mathrm{out}}}(k)T_{\mathrm{out}}(k)P_{\mathrm{man}}(k{)]}^{T} \end{array}$$

where $P_{\mathrm{man}}(k)$ is intake manifold pressure and $e_{T_{\mathrm{out}}}(k)$ is output torque tracking error defined as follows

(10) $$\begin{array}{c} e_{T_{\mathrm{out}}}(k)=T_{\mathrm{out},r}(k)-T_{\mathrm{out}}(k) \end{array}$$

where $T_{\mathrm{out},r}(k)$ is requested load reference.

To achieve the control objective, and output torque error, its derivatives, the FQ and $NO_{x}$ values, are added to the reward function, r(k+1) (equation (11)). Here, the agent will be penalized when the system produces more than 500 ppm $NO_{x}$ . The DDPG agent is designed to minimize load tracking error, engine-out $NO_{x}$ , and fuel consumption by maximizing the following reward function

(11) $$\begin{array}{c} r(k+1)=-(k_{1}e_{T_{\mathrm{out}}}(k)+k_{2}\frac{e_{T_{\mathrm{out}}}(k)-e_{T_{\mathrm{out}}}(k-1)}{T_{s}}\\ \;\;\;\;\;\;\;\;\;\;\;\;\;\;+k_{3}\mathrm{FQ}(k)+k_{4}NO_{x}(k)+k_{5}(NO_{x}(k)>500) \end{array}$$

where r(k) is reward and $T_{s}$ is the sampling time; in this application, it is each engine cycle or 0.08 s at a constant engine speed of 1500 r/min. $k_{1}$ to $k_{5}$ represents positive integer reward weights.

Figure 2 shows the network structure where the actor has nine fully connected layers (FCs) with a layer size of 64. The critic has 12 FCs with the same layer size (64) as the actor in each layer. The activation function of both the critic and actor FCs is rectified linear unit (ReLU) other than the output layers (FC12 in critic and FC9 in actor). The scaling layer is used in the output layers to standardize the output values. To train both the DDPG and safe DDPG, a mini-batch size of 64 and a smoothing factor of 0.001 are used. For training RL, the Adam optimizer with a learn rate of 0.0001 is used. A noise model has been implemented with a variance of 5.66, 0.42, and 0.01 for FQ(k) , SOI(k) , and VGT(k) , respectively. To force the RL to explore a larger region, the variance decay rate is chosen as a small value $\left(10^{-6}\right)$ . The ESM and the implementation of a safety filter to enforce the provided constraints are also shown in Figure 2.

### Safe RL versus RL

**Figure 3.**
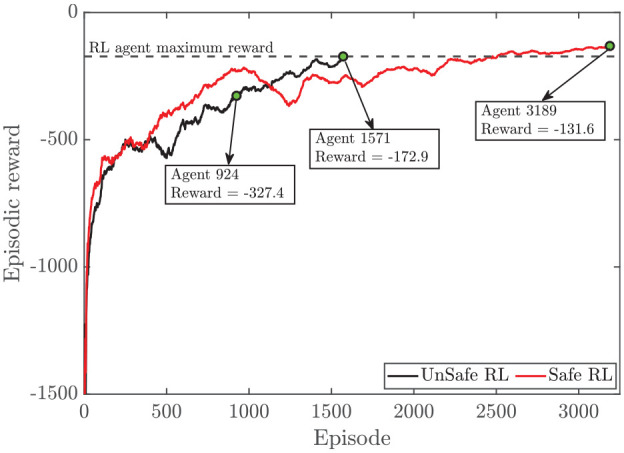
Episodic reward versus episode for safe RL and RL. Dashed line shows maximum episodic reward for RL agent to highlight the difference between RL and safe RL episodic reward.

In this study, two agents have been developed, a traditional DDPG implementation, called RL, and a DDPG with a safety filter to constrain the output, called safe RL. In both agents, the structure of actor and critic are kept same. The episodic reward that the agent receives versus the episode number is shown in Figure 3. A 40 s simulation (500 engine cycles) with a random load request, $T_{\mathrm{ref}}(k)$ , is provided to the agent in which the load reference is randomly changed for each episode. On an Intel Core i7-6700K–based PC with 32.0 GB RAM, running each episode takes an average of 346.84 s for the total ESM simulation and RL algorithm to update the networks. For the training of both agents, the simulation is run to a maximum of 5000 episodes. Both RL agents are run with different initialization of both the critic and actor networks over different random seeds. The best RL and best safe RL networks are chosen based on their maximum final reward value, as shown in Figure 3. These two agents are selected as they represent the best agents when compared to all of the saved agents with a reward higher than −150. Due to file size constraints, only agents with a reward higher than −150 were saved and then compared to select the agent that had the maximum reward. As shown in Figure 3, safe RL takes almost two times longer to reach the maximum reward compared to regular RL. This is due to the fact that the safe RL has more space which needs to be explored. Additionally, due to the use of a safety filter in safe RL, it reaches a larger reward which can be seen by comparing the agent at episode 1572 of RL and the agent at episode 3189 of safe RL (dashed line in Figure 3 is used to highlight this comparison).

**Figure 4.**
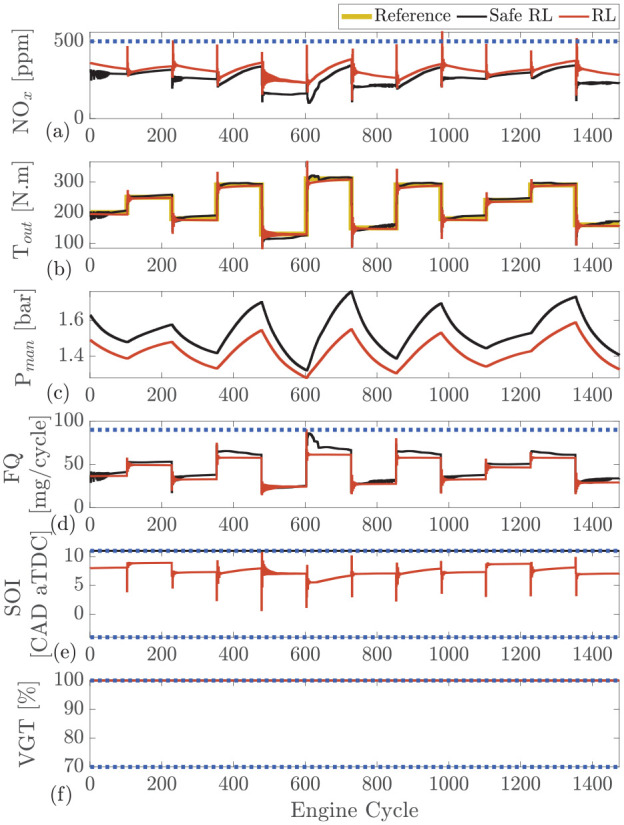
Safe RL versus RL: comparison between two agents that reach to maximum reward for safe RL (agent 3189 in Figure 3) and RL (agent 1571 in Figure 3) at engine speed of 1500 r/min. (a) engine-out $NO_{x}$ , (b) intake manifold pressure $\left(P_{\mathrm{man}}\right)$ , (c) engine output torque $\left(T_{\mathrm{out}}\right)$ , (d) fuel quantity (FQ) , (e) start of injection (SOI) , (f) variable geometry turbine (VGT) rate.

The comparison between the selected agents for both the safe RL and RL is presented in Figure 4. As shown, regardless of the training process, both agents are capable of maintaining load and minimizing $NO_{x}$ emissions and FQ. Even the RL tries to obey the constraints as they are included in the reward function. According to the results presented, the safety filter is not useful in final episode, and without the safety filter, RL can learn the constraints as well as minimize the tracking error and $NO_{x}$ .

**Figure 5.**
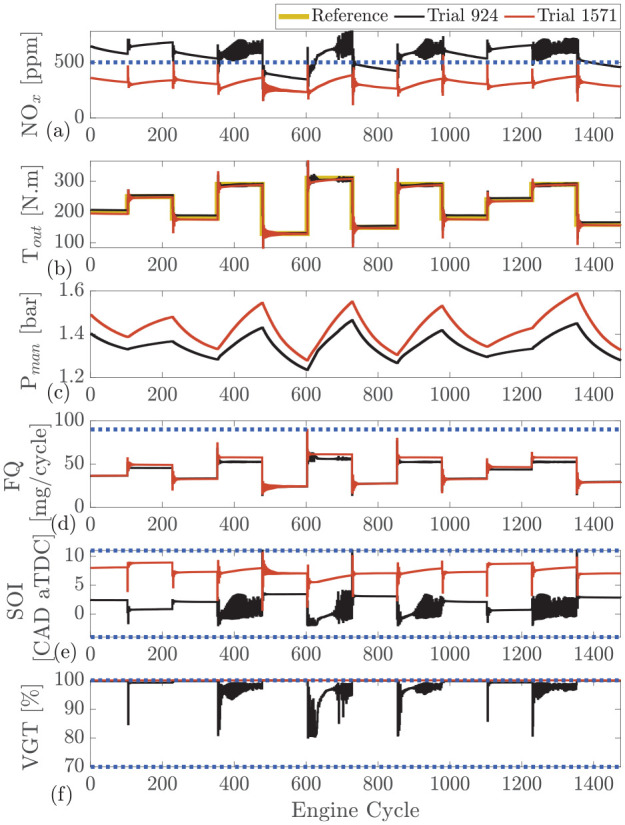
RL during training: comparison between agent in middle of training (agent 947 in Figure 3) and agent that reaches to maximum reward (agent 1571 in Figure 3) at engine speed of 1500 r/min. (a) engine-out $NO_{x}$ , (b) intake manifold pressure $\left(P_{\mathrm{man}}\right)$ , (c) engine output torque $\left(T_{\mathrm{out}}\right)$ , (d) fuel quantity (FQ) , (e) start of injection (SOI) , (f) variable geometry turbine (VGT) rate.

As shown in Figures 4 and 5, the NO_x_ emissions spike following each step. This is a result of SOI oscillating at the beginning of each cycle and jumping to advance combustion for a couple of engine cycles. Therefore, in those engine cycles, an increased amount of NO_x_ is formed.

The two final selected agents perform well; however, a more interesting comparison can be made during the training of the agents. Figure 5 shows the two agents of the RL during training. These agents are also presented in Figure 3. One agent is in the middle of the training process at episode 924 and the other is the final agent that has reached the maximum reward at episode 1571. The oscillation observed from the controller during the early stages of training (episode 924) is due to the white noise used to excite the system to allow for increased learning. When compared to agent 924, the fully trained agent 1571 is significantly better at observing all constraints. For the $NO_{x}$ output, the fully trained agent 1571 only exceeds the imposed limit for two cycles. For online training, the presence of safety filter is crucial in observing the constraints throughout training. However, if training is carried out in simulation, the use of a safety filter is not necessary, as the final agent is able to meet constraints while providing a stable output without the increased training time of using a safety filter.

## ILC

**Figure 6.**
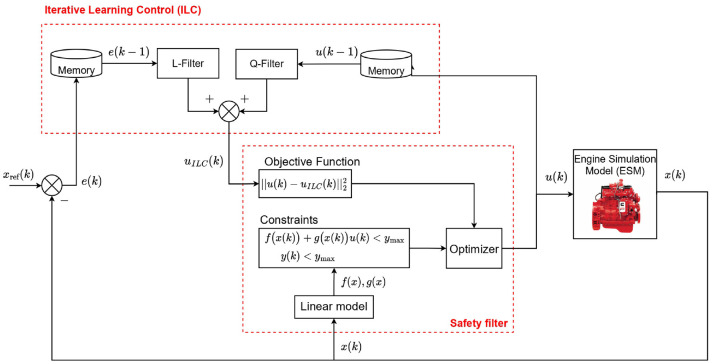
Safe iterative learning control block diagram.

One of the fast learning–based controllers that has common elements with RL is ILC. ILC has a simpler structure than RL as its control law update includes two main filters and can be defined as follows

(12) $$u_{t}(k)=Q(u_{j}(k-1))+L(e_{j}(k-1))$$

where $L(e_{j}(k))$ is the L-filter or learning filter and $Q(u_{j}(k))$ is the Q-filter . In this equation, k represents the time interval. One of the simplest types of ILC is P-type ILC where the learning filter is $Pe_{j}(k)$ and Q-filter is identity matrix where P is a proportional gain. Similar to safe RL, to enforce the output constraints, a safety filter is added to ILC. Figure 6 shows a block diagram of the safe ILC. As shown, ILC learns from the previous error and control input to generate the current control action.

**Figure 7.**
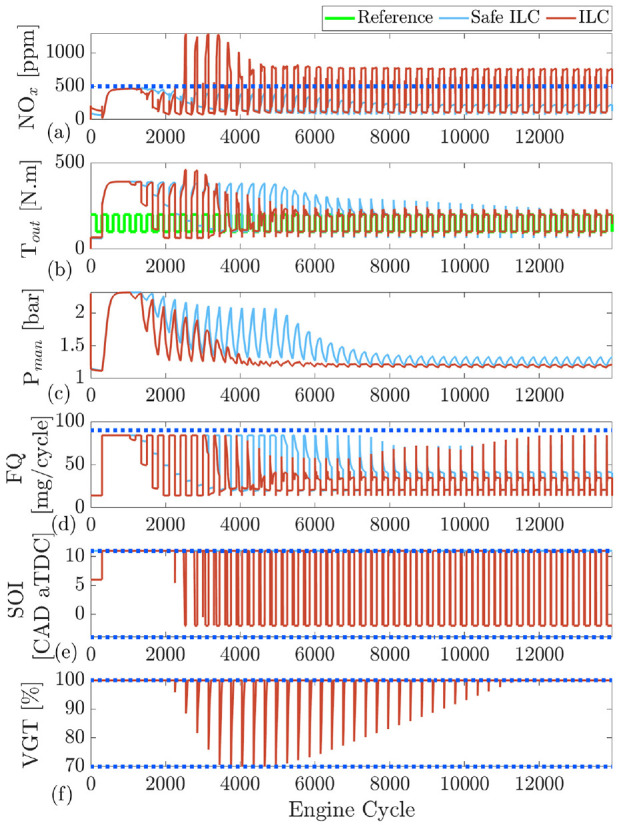
Training ILC and safe ILC at engine speed of 1500 r/min: reference is repeated every 300 cycles and 46 ILC iterations are shown: (a) engine-out $NO_{x}$ , (b) intake manifold pressure $\left(P_{\mathrm{man}}\right)$ , (c) engine output torque $\left(T_{\mathrm{out}}\right)$ , (d) fuel quantity (FQ) , (e) start of injection (SOI) , (f) variable geometry turbine (VGT) rate.

For implementation purposes, this is slightly different compared to RL. Because of the nature of repetitive input requirements, a repetitive reference has been implemented and the error between the actual state and reference is provided to the ILC. The error can be defined as follows

(13) $$\begin{array}{c} e(k)=\left[\begin{array}{c} NO_{x,\mathrm{ref}}(k)-NO_{x}(k)\\ T_{\mathrm{out},\mathrm{ref}}(k)-T_{\mathrm{out}}(k)\\ P_{\mathrm{in},\mathrm{ref}}(k)-P_{\mathrm{ref}}(k) \end{array}\right] \end{array}$$

where $NO_{x,\mathrm{ref}}(k)$ , $T_{\mathrm{out},\mathrm{ref}}(k)$ , and $P_{\mathrm{in},\mathrm{ref}}(k)$ are the respective reference values where each of these references are repetitive with the same frequency. As the only tracking problem is the load output from the engine, this reference is the actual reference and the other two are implemented to satisfy the repetition requirements. For $NO_{x}$ , the reference value changes from 20 to 40 ppm for minimizing it (instead of simply putting 0, a small variation is required). Similarly, a reference for intake manifold pressure the set point is changed from 2 to 2.1 bar. All of the references are repeated every 300 cycles, that is, for $NO_{x}$ , the set point is 20 ppm for 150 cycles, then changes to 40 ppm for 150 cycles and then repeated. ILC and safe ILC training are shown in Figure 7. This figure presents 46 ILC iterations (total of 13,800 engine cycles). As shown after cycle 33 (9900 engine cycle), both the safe ILC and ILC learn to track desired references. As shown, the safe ILC is able to observe the output constraints; however, the ILC fails to remain within the constraints. Here, unlike the RL implementation, the presence of a safety filter for both the final stage and during training is necessary. As shown, the safe ILC tends to require late injections as SOI remains saturated at the upper limit. Here, the existence of upper limit is necessary to avoid very late injection timing.

## Results and discussions

In this section, the two developed controllers, safe RL and safe ILC, will be compared to a previously developed LSTM-NMPC. The NMPC controller previously developed in the work by Norouzi et al.^59^ is used to compare the RL controllers here with MPC controller. All developed controllers are compared to a Cummins-calibrated ECU which modeled the simulation environment with the DPM and denoted “Benchmark (BM).”

**Figure 8.**
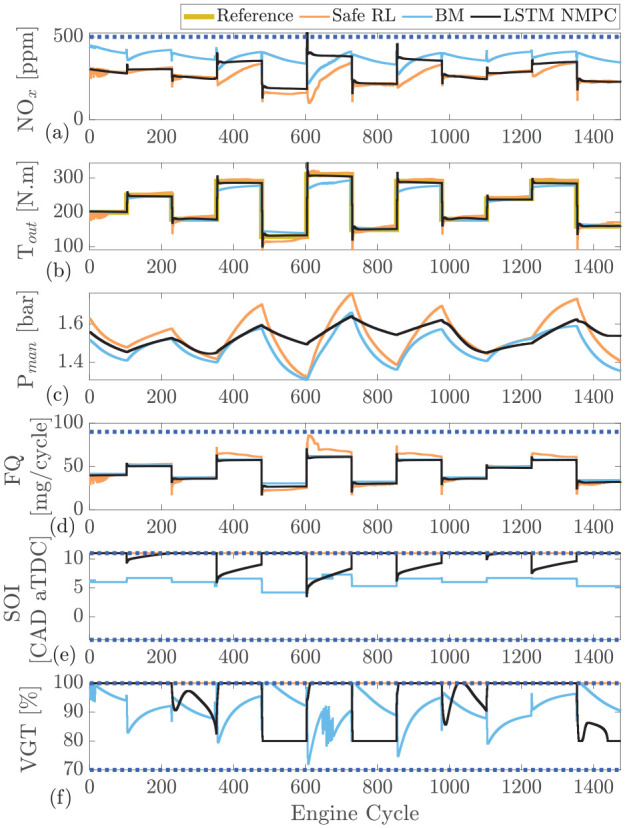
Safe reinforcement learning compared with long-short-term memory based nonlinear model predictive controller (LSTM-NMPC)^59^ and Cummins-calibrated ECU which is modeled in GT-Power at engine speed of 1500 r/min: (a) engine-out $NO_{x}$ , (b) intake manifold pressure $\left(P_{\mathrm{man}}\right)$ , (c) engine output torque $\left(T_{\mathrm{out}}\right)$ , (d) fuel quantity (FQ) , (e) start of injection (SOI) , (f) variable geometry turbine (VGT) rate.

The comparison between the RL, LSTM-NMPC, and BM controllers is presented in Figure 8. Both controllers solve a similar optimization problem but the reward function in RL has a slightly different cost function compared to the LSTM-NMPC. The cost function of LSTM-NMPC is defined as follows

(14) $$\begin{array}{c} J(u(\cdot |k),s(k))=\sum_{i=0}^{N_{p}-1}[\underset{\mathrm{Torque}\;\mathrm{output}\;\mathrm{tracking}}{\underset{︸}{||e_{T_{\mathrm{out}}}|{|}_{w_{T_{\mathrm{out}}}}^{2}}}\\ \;\;\;\;\;\;\;\;\;\;\;\;\;\;\;\;\;\;\;\;\;\;\;+\underset{NO_{x}\;\mathrm{minimizing}}{\underset{︸}{||NO_{x}(k+i)|{|}_{w_{NO_{x}}}^{2}}}+\underset{\mathrm{Fuel}\;\mathrm{consumption}\;\mathrm{minimizing}}{\underset{︸}{||\mathrm{FQ}(k+i)|{|}_{w_{\mathrm{FQ}}}^{2}}}\\ \;\;\;\;\;\;\;\;\;\;\;\;\;\;\;\;\;\;\;\;\;\;\;+\underset{\mathrm{Control}\;\mathrm{effort}\;\mathrm{penalty}}{\underset{︸}{||u(k+i|k)-u(k+i-1|k)|{|}_{w_{\Delta u}}^{2}}}\\ \;\;\;\;\;\;\;\;\;\;\;\;\;\;\;\;\;\;\;\;\;\;\;+\underset{\mathrm{Constraint}\;\mathrm{violation}\;\mathrm{penalty}}{\underset{︸}{w_{s}s{\left(k\right)}^{2}}}] \end{array}$$

where

(15) $$\left||.|{|}_{w}^{2}=[.{]}^{T}w[.\right]$$

where s(k) is a slack variable that is added for penalizing possible constraint violations.^59^ By comparing the LSTM-NMPC cost function (equation (14)) and the RL reward function (equation (11)), the torque tracking, fuel consumption, and $NO_{x}$ minimization are the same. In RL, the goal is maximizing the reward function and thus the negative sign is the reward function. The main difference between RL and NMPC in the reward/cost function is δu term in the NMPC that is added to resolve the oscillatory response of NMPC. The same relative weights between different terms of cost/reward function have been implemented to keep the tuning of the two controllers similar.

As shown in Figure 8, the safe RL is capable of accurately tracking the output torque with similar performance to the LSTM-NMPC. Both controllers outperform the BM feedforward production controller. Here the safe RL controller suffers from slightly increased overshoot when compared to the NMPC.

The controllers maintain $NO_{x}$ emissions levels below the defined 500 ppm $NO_{x}$ constraint. One clear trend in both the NMPC and RL is that the average $NO_{x}$ value is significantly lower than the BM. This is expected as both controllers minimize $NO_{x}$ and fuel consumption. One interesting trend is that the $NO_{x}$ emissions of the safe RL controller follow a similar trend to the BM but at a lower level. When comparing the RL to the NMPC, overall, the $NO_{x}$ emissions are generally below the NMPC values and a significant reduction can be seen during the couple cycles after a change in load where NMPC controller focuses on the load change resulting in a spike of $NO_{x}$ emissions.

Comparing the controllers, the values of cumulative $NO_{x}$ , FQ, and execution time are compared in Table 2. For determining the execution time of the NMPC, an open-source package acados^64,65^ is used for implementation. For the execution time, the idea is to examine the feasibility for real-time implementation and thus the deployment time of RL is only considered and the training time has been excluded. In this study, RL has almost three times faster execution time than the online NMPC optimization.

**Table 2.** Comparison between safe RL, Benchmark (BM), and nonlinear model predictive control.^59^

Controller	Cumulative $NO_{x}$ (ppm)	Average $NO_{x}$ (ppm)	Load error (%)	Cumulative FQ (g)	Average	Execution time (ms)^a^
					FQ (mg)	
BM	5.6 $\times 10^{5}$	376.8	3.95	67.9	46.0	–
Safe RL	3.8 $\times 10^{5}$	260.4	3.85	69.1	46.8	4.5
LSTM-NMPC^59^	4.3 $\times 10^{5}$	290.2	1.90	65.6	44.4	12.20^b^

FQ: fuel quantity; RL: reinforcement learning; LSTM-NMPC: long-short-term memory-based nonlinear model predictive controller.

a Per engine cycle of simulation.

b Average acados execution time.

As shown, RL has significantly lower $NO_{x}$ in comparison with both the BM and NMPC. The drawback of the RL controllers is slightly increased load error and FQ. However, the improvement in $NO_{x}$ reduction using a RL controller is more significant than the loss in load error and FQ.

**Figure 9.**
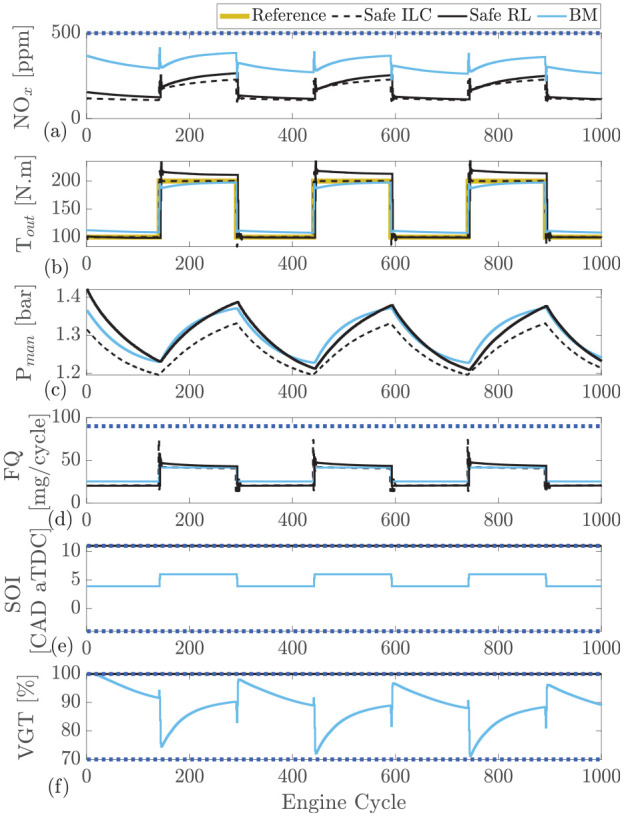
Safe reinforcement learning compared with safe ILC and Cummins-calibrated ECU which is modeled in GT-Power at engine speed of 1500 rpm: (a) engine-out $NO_{x}$ , (b) intake manifold pressure $\left(P_{\mathrm{man}}\right)$ , (c) engine output torque $\left(T_{\mathrm{out}}\right)$ , (d) fuel quantity (FQ) , (e) start of injection (SOI) , (f) variable geometry turbine (VGT) rate.

The safe RL controller performs comparably to the NMPC; however, it is also of interest to compare with another learning control strategy such as ILC. The developed safe RL controller is compared to safe ILC and the BM in Figure 9. As shown, both learning controllers are capable of tracking the desired output torque with similar performance to the BM. ILC tracks the reference more closely than safe RL control. The ILC tracking performance is almost perfect with very little overshoot which is one of the benefits of ILC since the repetitive input requirements allow the ILC learn by repetition. The RL controller suffers from slight torque overshoot but the performance is still acceptable.

All the controllers tested were able to remain below the defined 500 ppm $NO_{x}$ constraint. The $NO_{x}$ reduction using the ILC is slightly better than the RL controller, however, both controllers significantly outperform the BM. When comparing the controller in terms of FQ used, both the ILC and RL controllers use slightly less fuel than the BM. However, very little difference can graphically be seen between the RL and ILC controllers.

The controller performance results and the values of cumulative $NO_{x}$ , FQ, execution time are summarized in Table 3. The execution time is determined by measuring the turn around time of the controllers in Simulink where all controllers are testing on the same system. Around 75% of the execution time, on average, comes from solving the QP problem and the RL runs quite quickly as it evaluates a DNN (forward propagation). The QP solver in our simulation experiment shows the possibility of implementing this in the real-time experiment. This has been done in previous studies^59,66^ and good real-time performance has been achieved using acados^64,65^ solver.

**Table 3.** Comparison between safe RL, BM, and ILC.

	Cumulative $NO_{x}$ (ppm)	Average $NO_{x}$ (ppm)	Load error (%)	Cumulative FQ (g)	Average FQ (mg)	Execution time (ms)^a^
BM	3.17 $\times 10^{5}$	317.0	6.65	32.6	32.6	–
Safe RL	1.71 $\times 10^{5}$	171.0	5.23	31.4	31.4	4.5
Safe ILC	1.55 $\times 10^{5}$	155.4	0.51	29.9	29.9	0.08

FQ: fuel quantity; BM: Benchmark; RL: reinforcement learning; ILC: iterative learning controller.

a Per engine cycle of simulation.

As shown, both RL and ILC are able to reduce $NO_{x}$ emissions significantly compared to the BM. Although a comparison between the RL and ILC FQ showed a better FQ for ILC, the execution time of ILC is two orders of magnitude faster than the RL with significantly better load tracking performance. The fast learning time of the ILC indicates that it could be used for real-time online training. However, its main drawback is that it requires a repetitive reference or disturbance. This condition may be possible for stationary engines; however, it is not feasible in most of the ICE applications especially for on-road engines. Therefore, the slight performance loss of the RL compared to the ILC provides the flexibility to remove the requirement of a repetitive reference or disturbance.

## Summary and conclusion

A deep RL–based controller is developed to minimize the $NO_{x}$ emissions and fuel consumption of a diesel engine while tracking the required torque. Using a detailed ESM, a GT-Power/Matlab co-simulation of two learning-based controllers is investigated. The first is an RL controller utilizing a DDPG based on a deep network for both the actor and the critic. This is then extended with the addition of a safety filter. This safety filter is added to the manipulated control action and used to enforce output constraints. The second learning-based controller is based on ILC. The same safety filter is also applied to ILC to enforce the output constraints.

The learning-based controllers with safety filter are compared to their standard versions to better understand the effect of adding a safety filter. It was found that for deep RL, both the safe and standard controllers result in almost the same controller performance once training is completed. Even the standard RL is able to learn to enforce the output constraints. However, during training, there are large violations of the constraints suggesting that using safe learning is crucial when working with a real engineering system for real-time learning. For ILC, the safety filter implementation shows a significant effect during both training and final controller performance. This suggests that ILC requires a safety filter to enforce output constraints.

The safe RL is then compared to safe ILC to evaluate which controller has better performance as they both share a similar learning-based controller approach. This comparison shows that the deployment time of ILC is two orders of magnitude faster than RL and ILC has the ability to take advantage of online learning. Although ILC has a 4% better torque tracking and 16 ppm lower average $NO_{x}$ emissions than the RL-based controller, it is limited to repetitive references and disturbances. This makes ILC only feasible for stationary ICE applications which utilize a repetitive setpoint. However, few ICE applications are repetitive and thus using ILC for most on-road vehicle applications is not feasible.

To compare the safe RL to a state-of-the-art controller, a comparison is made to a model-based LSTM-NMPC.^59^ This comparison shows that the deep RL is capable of reducing the average $NO_{x}$ emissions by 30 ppm more than the LSTM-NMPC at a cost of 2% higher load error and 4.5% average fuel consumption increase. These performance differences between the models are very small. However, the LSTM-NMPC is a model-based controller which requires an accurate model for online MPC optimization. In contrast, the RL learns directly from experimental data but could violate constraints especially in training phase. Therefore, by adding a simple model as safety filter helps RL enforce output constraints. The summary of these comparisons is presented in Tables 4 and 5.

**Table 4.** Summary of comparison for developed controllers.

Method	Constraint enforcement	Execution time	Model requirement	Limitation
RL	–	50×	–	Time-consuming in training
ILC	–	1×	–	Repetitive reference requirement
Safe RL	✓	50×	✓	Time-consuming in training
Safe ILC	✓	1×	✓	Repetitive reference requirement
LSTM-NMPC^59^	✓	150×	✓	High-accuracy model requirement

RL: reinforcement learning; ILC: iterative learning controller; LSTM-NMPC: long-short-term memory-based nonlinear model predictive controller.

**Table 5.** Summary of comparison for developed controllers—controller performance compared to Benchmark.

Method	Load tracking error	Average fuel consumption reduction^a^	Average NO_x_ reduction^a^
Safe RL	3%−5%	0%−4%	30%−45%
Safe ILC	≤1%	≥8%	≥50%
LSTM-NMPC^59^	≤2%	≥3%	≥22%

RL: reinforcement learning; ILC: iterative learning controller; LSTM-NMPC: long-short-term memory-based nonlinear model predictive controller.

Range is used in safe RL as it is compared with BM using both repetitive and random reference twice with different reference.

a Reduction calculated relative to BM.

The application of using a safe learning–based control is demonstrated in simulation; however, for next-generation AI-powered engine controllers, these methods require extensive real-time data. Implementing either of these model-free learning-based controllers in real-time requires detailed testing on real hardware. Future work includes testing these methods on the engine in real time.
